# Insidious Transmission of a Stress-Related Neuroadaptation

**DOI:** 10.3389/fnbeh.2020.564054

**Published:** 2020-10-05

**Authors:** Jennifer S. Steger, Benjamin B. Land, Julia C. Lemos, Charles Chavkin, Paul E. M. Phillips

**Affiliations:** ^1^Center of Excellence in Neurobiology of Addiction, Pain and Emotion, University of Washington, Seattle, WA, United States; ^2^Department of Pharmacology, University of Washington, Seattle, WA, United States; ^3^Department of Psychiatry and Behavioral Sciences, University of Washington, Seattle, WA, United States; ^4^Department of Neuroscience, University of Minnesota, Minneapolis, MN, United States

**Keywords:** fast-scan cyclic voltammetry, CRF, dopamine, nucleus accumbens, stress, social stress, stress transmission

## Abstract

Stress is highly pervasive in humans, impacting motivated behaviors with an enormous toll on life quality. Many of the effects of stress are orchestrated by neuropeptides such as corticotropin-releasing factor (CRF). It has previously been shown that in stress-naïve male mice, CRF acts in the core of the nucleus accumbens (NAc) to produce appetitive effects and to increase dopamine release; yet in stress-exposed male mice, CRF loses its capacity to modulate NAc dopamine release and is aversive. In the current research, we tested whether this effect is comparable in females to males and whether the neuroadaptation is susceptible to social transmission. We found that, like in males, CRF increased dopamine release in stress-naïve but not stress-exposed female mice. Importantly, this persistent physiological change was not accompanied by overt behavioral changes that would be indicative of depression- or anxiety-like phenotype. Nonetheless, when these mice were housed for 7 days with stress-naïve conspecifics, the cage mates also exhibited a loss of dopamine potentiation by CRF. These data demonstrate the asymptomatic, yet pervasive transmission of stress-related neuroadaptations in the population.

## Introduction

The biological stress response is an intricate and tightly orchestrated adaptation that has presumably been influenced by natural selection to enhance the ability of organisms to cope with novel situations that require action or defense. The neuropeptide corticotropin-releasing factor (CRF) is released in the brain in response to stress (Cook, 2004; Merali et al., 2004; Wang et al., 2005; Ohmura et al., 2009; Holly et al., 2016), where it can activate the HPA axis (Vale et al., 1981; Rivier and Vale, 1983) and/or act at its centrally-expressed receptors CRFR1 and CRFR2 (Steckler and Holsboer, 1999; Van Pett et al., 2000; Sierra et al., 2015; Henckens et al., 2016) to direct a vast array of adaptive and maladaptive behavioral responses (Hostetler and Ryabinin, 2013). Previous studies demonstrate that, through its actions in the nucleus accumbens (NAc) core, CRF can promote appetitive behaviors (Peciña et al., 2006; Lim et al., 2007; Lemos et al., 2012) and increase dopamine release (Lemos et al., 2012, 2019). However, these effects are modulated by a prior stress experience: following exposure to a stressor, CRF loses the ability to increase mesolimbic dopamine release (Lemos et al., 2012, 2019) and produces an aversive behavioral response (Lemos et al., 2012). Importantly, experience-dependent dysregulation of the CRF system has been posited as a major contributor to vulnerability for stress hyperresponsivity as well as addiction- and depression-like behavior (Curtis et al., 1999; Liu et al., 2005; Beckstead et al., 2009; Lemos et al., 2012).

Stress vulnerability has a high degree of sexual dimorphism, with females being generally more stress-sensitive than males to psychiatric pathologic sequelae (Bale and Epperson, 2015; Hodes and Epperson, 2019). This increased vulnerability has been attributed to neuroendocrine effects in women, at least in part, because it can change across the estrous cycle (American Psychiatric Association, 2013). Moreover, the sexually dimorphic effects of stress on mental health are not simply quantitative (i.e., males and females show the same pattern of traits but to different extents) but also qualitative (i.e., a process varies fundamentally by sex or is only present in one sex; Beltz et al., 2019). For instance, whereas stress is more likely to trigger the onset of depressive disorders in females than males (Kessler et al., 2003), it is more likely to precipitate substance use disorders in males than females (Mchugh et al., 2018). Concerning CRF function, sex differences are observed in the expression of CRF peptide (Duncko et al., 2001; Viau et al., 2005; Iwasaki-Sekino et al., 2009; Sterrenburg et al., 2012), and the trafficking and signal-transduction coupling of CRF receptors (Bangasser et al., 2010; Valentino et al., 2013). Collectively, these sexually dimorphic effects indicate that the identification of neural mechanisms underlying stress-induced psychopathological traits in males has limited predictive capacity for females.

Additionally, stress vulnerability can extend beyond individuals who directly engage with a conventional stressor. Indeed, stress effects can be broadcast between domestic partners (Bolger et al., 1989). This stress transmission between individuals in a population is a phenomenon that has also been documented in non-human animals wherein behavioral, endocrine, and/or physiological changes have been engendered in conspecifics of stressed individuals (Zalaquett and Thiessen, 1991; Langford et al., 2006; Bruchey et al., 2010; Jeon et al., 2010; Bartal et al., 2011; Burkett et al., 2016; Sterley et al., 2018).

Here, we used fast-scan cyclic voltammetry in acute NAc slices to explore these factors in the interaction between CRF and dopamine in the NAc core of C57BL/6 mice. We demonstrate that the CRF-dopamine interactions in the NAc core are qualitatively similar between sexes where CRF potentiates dopamine release in both stress-naïve males and females but fails to do so following repeated forced swim stress (rFSS) in either sex. Moreover, the loss of CRF regulation of dopamine release is also exhibited in mice that are naïve to direct stress exposure but housed with animals that were subjected to rFSS.

## Materials and Methods

### Animals

Male and female C57BL/6 mice between 6 and 24 weeks of age were maintained under a 12-h light-dark cycle with access to standard food and water *ad libitum*. All procedures on animal subjects were approved by the University of Washington Institutional Animal Care and Use Committee. Stress-naïve or directly stressed mice were singly or group-housed (up to five same-sex mice per cage). Direct stress (donors) and indirectly stressed mice were housed in groups of four with two animals belonging to each group.

### Fast-Scan Cyclic Voltammetry

Mice were quickly decapitated, and the head was placed in pre-oxygenated ice-cold sucrose-based artificial cerebrospinal fluid (aCSF) solution (in mM: 248.3 sucrose, 3 KCl, 2 Mg_2_SO_4_.7H_2_O, 1.3 NaH_2_PO_4_ monobasic, 10 D-glucose anhydrous, 26 NaHCO_3_, 0.1 CaCl_2_ dihydrate). The brain was rapidly removed and blocked to isolate the anterior forebrain. This block of tissue was secured by the caudal surface to the specimen disc in the vibratome (Leica VT1000 S Vibratome, RRID:SCR_016495) buffer tray using Loctite Super Glue Gel Control. Coronal slices (250 μm) containing the NAc (+0.62–1.78 mm rostral to Bregma) were prepared in oxygenated ice-cold sucrose-aCSF. Prepared slices were then transferred to a holding chamber in a water bath maintained at 32–35°C for a minimum of 50 min. The holding chamber contained oxygenated (non-sucrose) aCSF (in mM: 124.1 NaCl, 3 KCl, 2 Mg_2_SO_4_.7H_2_O, 1.3 NaH_2_PO_4_ monobasic, 10 D-glucose anhydrous, 26 NaHCO_3_, 2.5 CaCl_2_ dihydrate). After 50 min, the holding chamber was removed from the water bath and left at room temperature for an additional 10 min before slices were used for the experiment. Slices were placed in a recording chamber and continuously perfused (1.5–2.0 ml/min) with oxygenated aCSF maintained at 31–33°C. Fused-silica-insulated carbon-fiber microelectrodes (Clark et al., 2010), fabricated in-house, were inserted just ventral or ventrolateral to the anterior commissure (within 250 μm) inside the boundaries of the NAc core subregion (slices between +0.74 and 1.78 mm rostral to Bregma were used for recordings with a majority of recordings occurring in slices +1.10 to 1.34 mm rostral to Bregma). The potential at a carbon-fiber microelectrode was held at −0.4 V vs. Ag/AgCl, ramped to +1.3 V, and back to −0.4 V (400 V/s) every 100 ms. A single biphasic electrical pulse (2 ms per phase, 120–200 μA) was applied to the slice to evoke dopamine release every 2 min. Data were collected using TarHeel CV (University of North Carolina, Chapel Hill, NC, USA). Once a stable baseline was established with four consecutive dopamine recordings within 10% of each other, CRF (100 nM or 1 μM) or vehicle was administered for 30 min. Each brain slice used from an individual animal was assigned to a different treatment (i.e., replicates represent inter-subject variability). Across treatment groups there were no baseline differences in the absolute dopamine concentration evoked by stimulation (two-way ANOVA with Bonferroni’s *post hoc* tests; interaction of Pretreatment × Sex, *F*_(2,100)_ = 0.2758, *P* = 0.7596; Supplementary Figure S1A) nor were there differences in the stability of evoked release following vehicle treatment (two-way RM ANOVA with Bonferroni’s *post hoc* tests; stress-naive: interaction of Time × Sex, *F*_(15,255)_ = 1.166, *P* = 0.2984; direct stress: interaction of Time × Sex, *F*_(15,120)_ = 0.7500, *P* = 0.7292; indirect stress: interaction of Time × Sex, *F*_(15,150)_ = 1.037, *P* = 0.4210; Supplementary Figures S1B–D).

### Repeated Forced Swim Stress

Mice were subjected to a 2-day, modified Porsolt forced swim stress (Porsolt et al., 1977) as described previously (McLaughlin et al., 2003). On Day 1, animals were placed in a vessel of 30–32°C water for 15 min. Twenty-four hours later (Day 2), animals underwent four 6-min swims separated by 6-min recovery sessions in their home cage. Animals were then returned to their home cage for 7 days before voltammetry or behavioral testing was conducted. Overhead video footage of all swim trials was recorded using a Canon ZR90 camcorder and time spent immobile in the first 5 min of each swim session was analyzed using EthoVision (version 3.0; Noldus Information Technology, RRID:SCR_004074) with the default immobility threshold of 20%.

### Behavioral Tests

Mice were handled daily on the 3 days preceding behavioral testing. Each subject participated in a single behavioral test [i.e., three-chamber social approach, tail suspension test (TST), or elevated plus maze (EPM)]. At least 1 h before the start of testing, mice were moved to the procedure space, located close to their housing, and allowed to acclimate. Mice were returned to their home cage once all cage mates had been tested. The three-chamber social approach, TST, and EPM behavioral tests are described in detail below.

### Three-Chamber Social Approach

Social preference was assayed using the three-chamber social approach assay as described previously (Stein et al., 2019). Twenty-four hours before the beginning of the experiment, sex- and age-matched target (novel) mice were habituated to being enclosed in an inverted wire pencil cup (10.16 cm width × 10.16 cm depth × 10.8 cm height, Galaxy Pencil and Utility Cup; Spectrum Diversified Designs, LLC) for 1 h in an open field (OF) box. A custom-built white opaque acrylic three-chambered apparatus (62.23 cm length × 31.75 cm width × 31.12 cm height with two transparent internal partitions measuring 15.24 cm high × 29.21 cm wide, 5.08 cm × 5.08 cm square openings in the center to allow for travel in between the three chambers; TAP Plastics, Seattle, WA, USA) was utilized for these studies. Following a 10-min habituation period, in which the experimental mouse was placed into the center chamber and allowed to freely explore the apparatus, the mouse was briefly removed and placed in a holding cage. Two pencil cups were inverted and placed in the far corners of the apparatus. One cup remained empty and a target mouse was placed in the other inverted cup. Weighted cylindrical bottles were placed atop the inverted cups to prevent the experimental mouse from climbing on top of said cups. The experimental mouse was subsequently reintroduced into the center chamber and was free to explore the apparatus for an additional 10 min. Target mice were not used for consecutive trials and each target mouse was used for no more than four trials in 1 day. The side of the chamber that contained the target mouse was counterbalanced between trials and the pencil cups and the apparatus were cleaned with 35% ethanol and paper towels in between each trial. The experimental mouse’s movement was recorded with an HD ceiling-mounted camera (Panasonic WV-CP504). Time spent in each chamber and time spent in a proximal circle extending 9 cm beyond each of the wire enclosures was recorded and analyzed using EthoVision XT (version 14.0; Noldus Information Technology, RRID:SCR_000441). Heatmaps of activity were generated using Ethovsion XT (version 11.0; Noldus Information Technology, RRID:SCR_000441).

### Tail Suspension Test (TST)

The TST was employed to evaluate depression-like behavior and was conducted as previously described (Can et al., 2012). Mice were suspended by their tails from one half of a conditioned-place preference (CPP) chamber constructed from clear acrylic (20 cm length × 20 cm depth × 20 cm height) and placed on its side, which was nested within a white acrylic OF box (40.6 cm length × 40.6 cm width × 30 cm height) placed on its side. The CPP–OF box setup was used to improve video contrast and facilitate mouse movement tracking. The CPP–OF apparatus was positioned at the edge of the lab bench (3 ft height) in the procedure room, such that when suspended by their tails, mice dangled over the side of the lab bench. Before the experiment, a hollow cylindrical tube used to ensheath the tail to impede tail climbing behavior once suspended and a piece of tape used to attach the tail to the CPP box were prepared for each experimental mouse. Specifically, a Falcon 3 ml Transfer Pipet (Corning Inc.) that had the bulb and tip cut off was trimmed down to a 4-cm long hollow cylindrical tube and a 13-cm long piece of VWR General-Purpose Laboratory Labeling Tape (12.7 mm width; VWR International, LLC) was marked with a permanent marker at 5.5 cm, 10 cm, and 11 cm relative to one end. To carry out the experiment, a mouse’s tail was passed through the hollow tube and the section of prepared tape between 10–11 cm was wrapped around the end of a mouse’s tail leaving 2–3 mm of the tail exposed at the end. Experimental mice were transported from their home cage in the palm of the experimenter’s hand and the tape position marked at 5.5 cm was attached to the top of the CPP box in the center, such that mice could not contact the sides of the CPP box. Mice were then suspended by their tails for the 6-min trial. The CPP–OF apparatus was cleaned with 70% ethanol and paper towels in between trials. Mouse movement was recorded with a Canon ZR90 camcorder affixed to a tripod to capture side-on immobility. Immobility was analyzed using EthoVision (version 3.0; Noldus Information Technology, RRID:SCR_004074) with an immobility threshold of 10%.

### Elevated Plus Maze (EPM)

EPM testing was conducted as previously described (Bruchas et al., 2009) with noted modifications. The EPM (38 cm arm length × 7.62 cm width × 75 cm height; Med Associates, Inc.) was constructed out of black acrylic, and the open and closed arms were outfitted with strips of laminated white paper (5.5 cm width) to enhance video contrast and mouse tracking. On the day of the experiment, the EPM position and lighting were adjusted to minimize shadows in the closed arms, and such that light detected at the ends of the open arms measured 15 lux. To conduct the experiment, the experimental mouse was transported from its home cage to the EPM in the palm of the experimenter’s hand. The mouse was gently placed in the center of the EPM facing a corner formed by an open and closed arm and allowed to freely explore the apparatus for 6 min. In between trials, the apparatus was cleaned with 70% ethanol and paper towels. Movements were video recorded using a Canon ZR90 camcorder and analyzed using EthoVision (version 3.0; Noldus Information Technology, RRID:SCR_004074) and heatmaps of activity were generated using Ethovsion XT (version 11.0; Noldus Information Technology, RRID:SCR_004074). We used open arm time expressed as a percentage of total time as the primary measure of anxiety-like behavior. A subset of the directly and indirectly stressed mice used in the EPM experiments were subsequently used for voltammetry experiments.

### Statistical Analysis

Statistical analyses were performed, and graphs were generated using Prism 8 (GraphPad Software Inc., RRID:SCR_002798). Details of statistical tests employed can be found in the text. *Post hoc* power calculations were conducted using G*Power 3.1.9.4 (Heinrich-Heine-Universität Düsseldorf, RRID:SCR_013726). Bonferroni and Dunnett *post hoc* tests were used. The Dunnett test was employed when comparing every experimental group mean to the control group mean and the Bonferroni test was employed when one of the following two criteria was met: (1) if the data table only had two columns (or rows); or (2) if the data table being analyzed had more than two columns (or rows) but was comparable to a dataset that only had two columns (or rows) that was analyzed using a Bonferroni *post hoc* test. Notably, using the Dunnett test did not change whether any dataset reached significance compared to using a Bonferroni test.

### Chemicals and Drugs

Sucrose and NaH_2_PO_4_ monobasic were obtained from Sigma–Aldrich. KCl, Mg_2_SO_4_.7H_2_O, Dextrose (D-glucose) anhydrous, CaCl_2_ dihydrate, NaCl, NaHCO_3_, and glacial acetic acid were obtained from Thermo Fisher Scientific. CRF (human, rat) was obtained from Tocris Bioscience (Catalog No. 1151). Concentrated stock solutions of CRF (100 μM or 1 mM) and its vehicle (7% acetic acid in molecular biology grade water) were prepared and stored at −20°C for up to 1 week. Concentrated stocks were thawed on ice before being pipetted directly into oxygenated aCSF. Final concentrations of CRF and its vehicles were 100 nM (from 100 μM) or 1 μM (from 1 mM) and 0.07% by volume, respectively.

## Results

**Figure 1:**
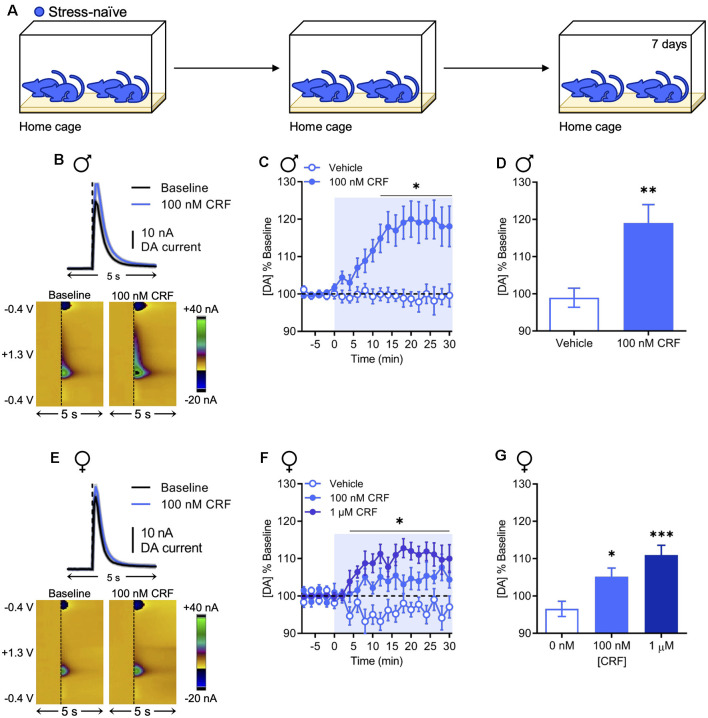
Corticotropin-releasing factor (CRF) increases dopamine release in the nucleus accumbens (NAc) in stress-naïve males and females. **(A)** In the stress-naïve experimental group, mice remained in their home cage for the duration of the experiment. **(B)** Representative dopamine release evoked by electrical stimulation (dashed line) before and after application of 100 nM CRF to a NAc brain slice collected from a stress-naïve male (mean ± SEM for five consecutive stimulations, top) and corresponding two-dimensional plots depicting changes in faradaic current (pseudocolor) with time as the abscissa and applied potential as the ordinate (bottom). **(C)** Baseline-normalized mean peak amplitude of electrically evoked dopamine release over time in response to vehicle (*n* = 9) or 100 nM CRF (*n* = 11) application to NAc slices from stress-naïve males. **(D)** Baseline-normalized mean peak amplitude of dopamine release 20–30 min after vehicle (*n* = 9) or 100 nM CRF (*n* = 11) application to NAc slices from stress-naïve males. **(E)** Representative dopamine release evoked by electrical stimulation (dashed line) before and after application of 100 nM CRF to a NAc brain slice from a stress-naïve female (mean ± SEM for five consecutive stimulations, top) and corresponding color plots (bottom). **(F)** Baseline-normalized mean peak amplitude of electrically evoked dopamine release over time in response to vehicle (*n* = 10), 100 nM CRF (*n* = 8), or 1 μM CRF (*n* = 8) application to NAc slices from stress-naïve females. **(G)** Baseline-normalized mean peak amplitude of dopamine release 20–30 min after vehicle (*n* = 10), 100 nM CRF (*n* = 8), or 1 μM CRF (*n* = 8) application to NAc slices from stress-naïve females. Error bars, SEM. *P < 0.05, **P < 0.01, ***P < 0.001 vs. vehicle.

To ascertain whether the effects of CRF on dopamine release in the NAc are sexually dimorphic, we selectively monitored dopamine release evoked by a single biphasic electrical pulse in acute coronal brain slices collected from stress-naïve animals (Figure 1A) using fast-scan cyclic voltammetry at carbon-fiber microelectrodes. Vehicle (0.07% acetic acid in artificial cerebrospinal fluid) or CRF (100 nM or 1 μM) was applied to the slice for 30 min after a stable baseline was achieved. In stress-naïve males, it has been shown that CRF increases NAc dopamine release in a concentration-dependent manner with maximal effects at 100 nM (Lemos et al., 2012). We replicated this effect with 100 nM CRF, which significantly increased dopamine release (Figure 1B) relative to vehicle beginning 12 min after drug application and persisting for the duration of the recording [two-way repeated-measures analysis of variance (RM ANOVA) with Bonferroni’s *post hoc* multiple comparisons tests; interaction of Time × Drug, *F*_(15,270)_ = 7.651, *P* < 0.0001; main effect of time, *F*_(15,270)_ = 6.374, *P* < 0.0001; main effect of the drug, *F*_(1,18)_ = 11.41, *P* = 0.0034; Figure 1C]. The resultant effect was quantified by averaging the evoked maximum dopamine current in the final 10 min of the recording (98.94% ± 2.57% and 119.1% ± 4.93% change from baseline in response to the vehicle or 100 nM CRF, respectively, mean ± SEM; unpaired *t*-test; *t*_(18)_ = 3.387, *P* = 0.0033; Figure 1D). The interaction between CRF and dopamine has not been previously examined in females. Therefore, we tested the effect of 100 nM and 1 μM CRF on NAc dopamine release. Similar to males, CRF increased NAc dopamine (Figure 1E) over time relative to vehicle treatment (two-way RM ANOVA with Bonferroni’s *post hoc* tests; interaction of Time × Drug, *F*_(28,322)_ = 1.792, *P* = 0.0095; main effect of time, *F*_(14,322)_ = 1.966, *P* = 0.0199; main effect of drug, *F*_(2,23)_ = 12.70, *P* = 0.0002; Figure 1F) and this effect was concentration-dependent (96.55% ± 2.05%, 97.52% ± 2.32%, and 111.0% ± 2.6% change from baseline in response to vehicle, 100 nM CRF, or 1 μM CRF, respectively, mean ± SEM; one-way ANOVA with Dunnett’s *post hoc* tests; *F*_(2,23)_ = 10.36, *P* < 0.001; Figure 1G). In NAc slices from females, the effect of 100 nM CRF appeared to be modest. However, when accounting for vehicle, comparison of this effect between sexes was not statistically significant (two-way ANOVA with Bonferroni’s *post hoc* tests; interaction of Sex × Drug, *F*_(1,34)_ = 2.750, *P* = 0.1064; Supplementary Figure S2). These data indicate that the effect of CRF on NAc dopamine release is qualitatively similar across sexes without significant quantitative sex differences.

**Figure 2:**
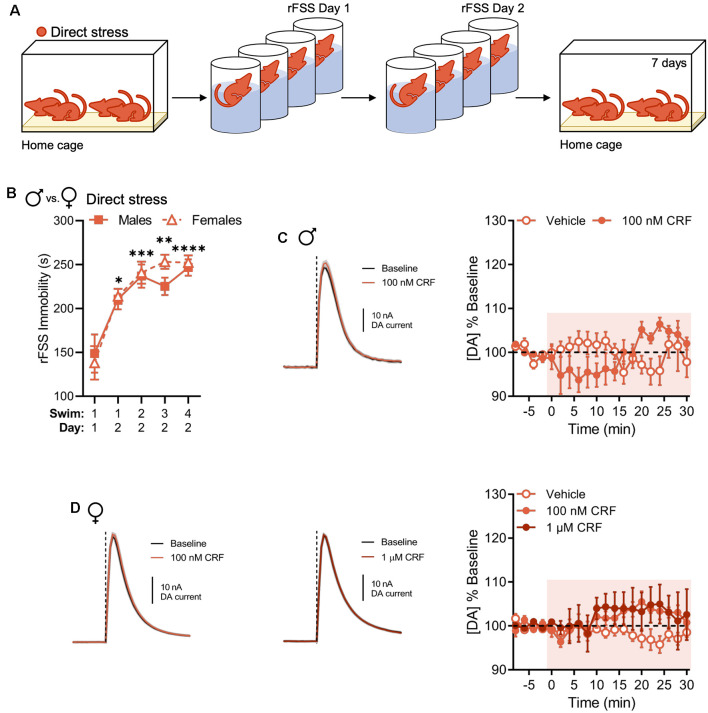
Direct stress disrupts CRF-mediated dopamine release in the NAc 1 week after repeated forced swim stress (rFSS) exposure in males and females. **(A)** In the direct stress experimental group, mice were exposed to rFSS and tested 7 days later. **(B)** Cumulative time spent immobile for the first 5 min of Swim 1 on rFSS Day 1 and Swims 1–4 on rFSS Day 2 in males (*n* = 6) and females (*n* = 10) belonging to the direct stress group. **(C)** Representative dopamine release evoked by electrical stimulation (dashed line) before and after application of 100 nM CRF to a NAc brain slice (mean ± SEM for five consecutive stimulations, left) and baseline-normalized mean peak amplitude of electrically evoked dopamine release over time in response to vehicle (*n* = 5) or 100 nM CRF (*n* = 5) application to NAc slices (right) from directly stressed males 1 week after stress exposure. **(D)** Representative dopamine release evoked by electrical stimulation before and after application of 100 nM CRF (left) or 1 μM CRF (middle) to a NAc slice (mean ± SEM for five consecutive stimulations) and baseline-normalized mean peak amplitude of electrically evoked dopamine release over time in response to vehicle (*n* = 5), 100 nM CRF (*n* = 5), or 1 μM CRF (*n* = 5) application to NAc slices (right) from directly stressed females 1 week after stress exposure. Error bars, SEM. *P < 0.05, **P < 0.01, ***P < 0.001, ****P < 0.0001 vs. rFSS Swim 1 Day 1 immobility.

In NAc slices from males, the effect of CRF on NAc dopamine release was previously found to be sensitive to prior rFSS experience (Lemos et al., 2012). In the present experiments, we tested whether this effect generalized to females using a direct stressor in the form of rFSS (Figure 2A). We observed escalating increases in rFSS immobility over swim sessions in all animals. This effect was not different between sexes (two-way RM ANOVA with Dunnett’s *post hoc* tests; interaction of Swim Session × Sex, *F*_(4,56)_ = 0.5415, *P* = 0.7059; Figure 2B). One week after direct rFSS exposure animals were evaluated for changes in CRF-induced modulation of evoked NAc dopamine release. In NAc slices from males, there was a significant interaction between time and drug in the absence of significant main effects of time or drug (two-way RM ANOVA with Bonferroni’s *post hoc* tests; interaction of Time × Drug, *F*_(15,120)_ = 3.606, *P* < 0.0001; main effect of time, *F*_(15,120)_ = 1.100, *P* = 0.3636; main effect of drug, *F*_(1,8)_ = 0.011, *P* = 0.9190; Figure 2C). This is a qualitative replication of previous findings (Lemos et al., 2012). Similarly, 100 nM or 1 μM CRF did not significantly increase evoked NAc dopamine in direct stress-exposed females over time relative to vehicle (two-way RM ANOVA with Bonferroni’s *post hoc* tests; interaction of Time × Drug, *F*_(30,180)_ = 1.169, *P* = 0.2632; Figure 2D). Therefore, we did not observe sexual dimorphism in this stress-related neurochemical adaptation.

**Figure 3:**
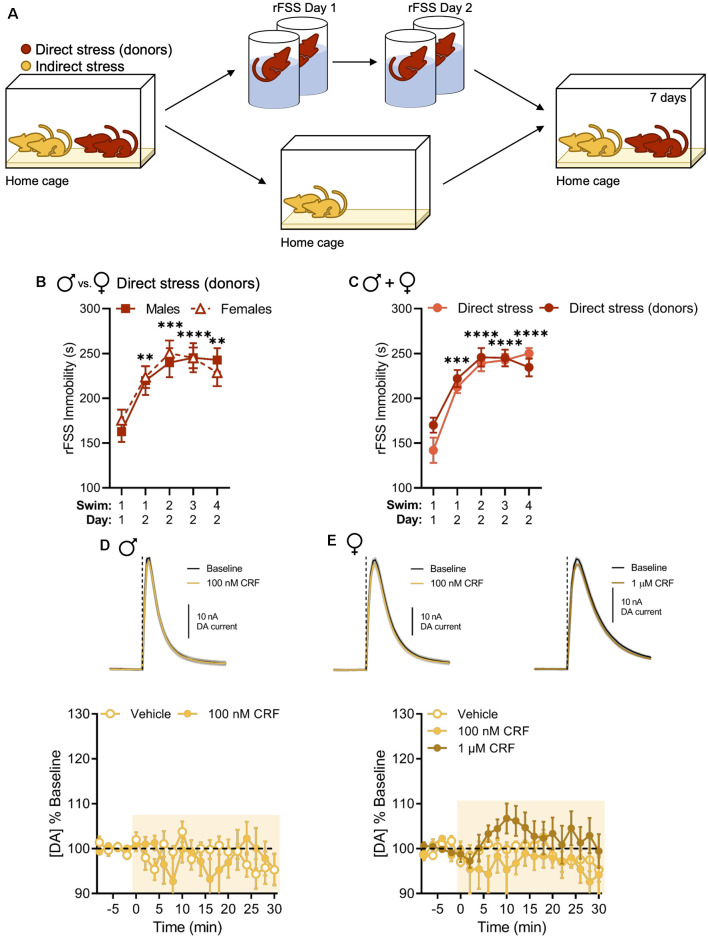
Indirect stress disrupts CRF-mediated dopamine release in the NAc in males and females 1 week after the onset of stress exposure. **(A)** Mice were housed in groups of four and two mice were subjected to direct stress. The two mice that did not undergo direct stress, termed the indirect stress group, remained in the home cage for the duration of the experiment. **(B,C)** Cumulative time spent immobile for the first 5 min of Swim 1 on rFSS Day 1 and Swims 1–4 on rFSS Day 2 in panel **(B)** males (*n* = 6) and females (*n* = 8) that were housed with indirectly stressed animals **(C)** mice (male and female groups combined) belonging to the direct stress (*n* = 16) and direct stress donors (*n* = 14) groups. **(D)** Representative dopamine release evoked by electrical stimulation (dashed line) before and after application of 100 nM CRF to a NAc brain slice (mean ± SEM for five consecutive stimulations, top) and baseline-normalized mean peak amplitude of electrically evoked dopamine release over time in response to vehicle (*n* = 4) or 100 nM CRF (*n* = 6) application to NAc slices (bottom) from indirectly stressed males 1 week after cage mates were directly stressed. **(E)** Representative dopamine release evoked by electrical stimulation before and after application of 100 nM CRF (top, left) or 1 μM CRF (top, right) to a NAc slice (mean ± SEM for five consecutive stimulations) and baseline-normalized mean peak amplitude of electrically evoked dopamine release over time in response to vehicle (*n* = 8), 100 nM CRF (*n* = 5), or 1 μM CRF (*n* = 8) application to NAc slices (bottom) from indirectly stressed females 1 week after cage mates were directly stressed. Error bars, SEM. **P < 0.01, ***P < 0.001, ****P < 0.0001 vs. rFSS Swim 1 Day 1 immobility.

Now that we replicated our previous findings in males and extended them to include females, our primary goal was to test whether the stress-related change in the regulation of NAc dopamine by CRF could be socially transmitted. Therefore, mice were housed with cage mates that underwent rFSS (Figure 3A). Again, we observed escalating increases in immobility over rFSS sessions in all the “donor” animals that were subsequently housed with the “indirect-stress” subjects. This effect was not different between sexes in the directly stressed group that was used to provide indirect stress (two-way RM ANOVA with Dunnett’s *post hoc* tests; interaction of Swim Session × Sex, *F*_(4,48)_ = 0.4469, *P* = 0.7741; Figure 3B). Moreover, no differences in swim immobility were observed between these and the previous direct stress groups (two-way RM ANOVA with Dunnett’s *post hoc* tests; interaction of Swim Session × Sex, *F*_(4,112)_ = 1.688, *P* = 0.1577; Figure 3C), indicating that cohousing with rFSS-naïve animals after the first stress session did not impact swim immobility behavior of stress “donors” on Day 2. Surprisingly, the CRF-dopamine interaction was also lost in indirectly stressed animals when evaluated 1 week after their cage mates were exposed to direct stress. This lack of effect was present in both males and females (two-way RM ANOVA with Bonferroni’s *post hoc* tests; males: interaction of Time × Drug, *F*_(15,120)_ = 1.428, *P* = 0.1449; females: interaction of Time × Drug, *F*_(30,270)_ = 1.150, *P* = 0.2761; Figures 3D,E). Thus, these data indicate that social transmission of the stress-related physiological adaptation had occurred in both sexes and, like with the direct stress effect, was not sexually dimorphic.

**Figure 4:**
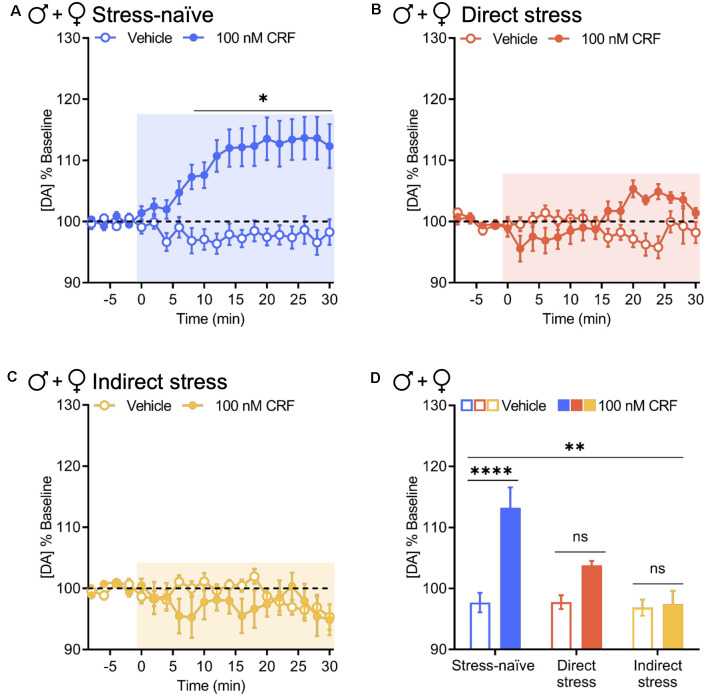
Direct and indirect stress disrupt CRF-mediated dopamine release in the NAc 1 week after stress exposure. Baseline-normalized mean peak amplitude of electrically evoked dopamine release over time in response to vehicle or 100 nM CRF application to NAc slices collected from panel **(A)** stress-naïve males and females (vehicle and 100 nM CRF: *n* = 19); **(B)** directly stressed males and females 1 week after rFSS exposure (vehicle and 100 nM CRF: *n* = 10); and **(C)** indirectly stressed males and females 1 week after cage mates were subjected to direct stress (vehicle: *n* = 12, 100 nM CRF: *n* = 11). **(D)** Baseline-normalized mean peak amplitude of dopamine release 20–30 min after vehicle or 100 nM CRF application to NAc slices from stress-naïve (vehicle and 100 nM CRF: *n* = 19), directly stressed (vehicle and 100 nM CRF: *n* = 10), or indirectly stressed (vehicle: *n* = 12, 100 nM CRF: *n* = 11) males and females. Error bars, SEM. ns: *P* > 0.05, *P < 0.05 vs. vehicle, **P < 0.01 vs. vehicle, ****P < 0.0001 for interaction.

When data from male and female groups were combined to increase power for statistical analysis, these results held up. CRF significantly increased evoked NAc dopamine release in stress-naïve animals over time relative to vehicle (two-way RM ANOVA with Bonferroni’s *post hoc* tests; interaction of Time × Drug, *F*_(15,540)_ = 6.686, *P* < 0.0001; main effect of time, *F*_(15,540)_ = 5.018, *P* < 0.0001; main effect of drug, *F*_(1,36)_ = 18.47, *P* = 0.0001; Figure 4A). Although we observed a significant interaction between time and drug in animals tested 1 week after direct and indirect stress exposure, there were no significant main effects of time or drug (two-way RM ANOVA with Bonferroni’s *post hoc* tests. Direct stress: interaction of Time × Drug, *F*_(15,270)_ = 5.525, *P* < 0.0001; main effect of time, *F*_(15,270)_ = 1.309, *P* = 0.1960; main effect of drug, *F*_(1,18)_ = 1.403, *P* = 0.2517. Indirect stress: interaction of Time × Drug, *F*_(15,315)_ = 1.740, *P* = 0.0426; main effect of time, *F*_(15,315)_ = 1.306, *P* = 0.1966; main effect of drug, *F*_(1,21)_ = 0.5198, *P* = 0.4789; Figures 4B,C). Directly comparing the resultant effects of 100 nM CRF vs. vehicle on evoked NAc dopamine release in the final 10 min of the recording between stress pretreatment groups, we found that relative to vehicle, CRF significantly increased NAc dopamine in stress-naïve mice (97.69% ± 1.60% and 113.22% ± 3.36%, respectively, mean ± SEM), but this effect was attenuated in direct stress- (97.77% ± 1.13% and 103.80% ± 0.73%, respectively, mean ± SEM) and indirect stress-exposed (96.88% ± 1.32% and 97.42% ± 2.2%, respectively, mean ± SEM) groups, resulting in a significant group by drug interaction (two-way ANOVA with Bonferroni’s *post hoc* tests; interaction of Stress Pretreatment × Drug, *F*_(2,75)_ = 5.690, *P* = 0.0050; main effect of pretreatment, *F*_(2,75)_ = 6.736, *P* = 0.0020; main effect of drug, *F*_(1,75)_ = 13.48, *P* < 0.001; Figure 4D). Thus, as assayed here, the consequences of stress were especially pervasive as not only did stress perturb a veritable neurochemical interaction in the animals that were directly exposed to rFSS, but also in their cage mates.

**Figure 5:**
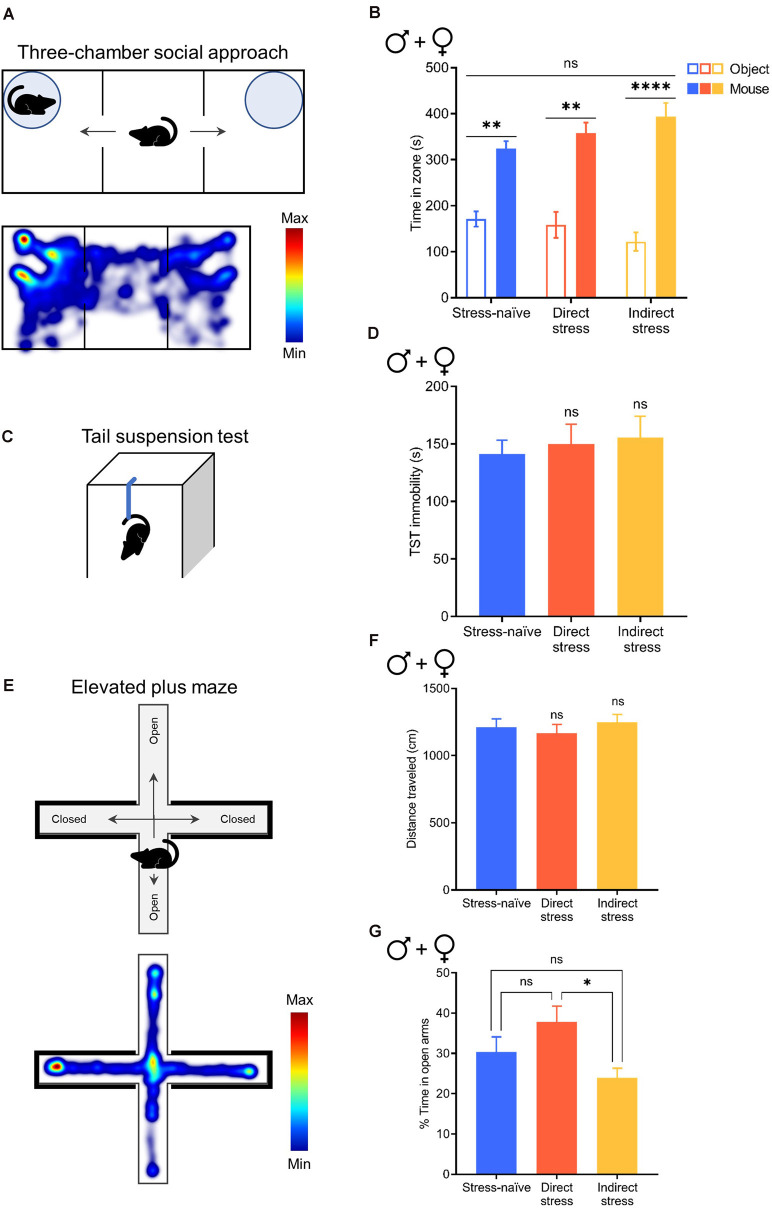
Direct and indirect stress do not affect social interaction, depression-like behavior, or anxiety-like behavior 1week after stress exposure. **(A)** Cartoon depicting the three-chamber social approach assay (top) and a representative heatmap of activity during this test (bottom). **(B)** Time spent in the novel object and novel mouse zones in the three-chamber social approach assay in stress-naïve (*n* = 8), directly stressed (*n* = 6), and indirectly stressed (*n* = 7) males and females. **(C)** Cartoon depicting the tail suspension test (TST). **(D)** Time spent immobile for the 6-min duration the TST in stress-naïve (*n* = 10), directly stressed (*n* = 11), and indirectly stressed (*n* = 10) males and females. **(E)** Cartoon depicting the elevated plus maze (EPM, top) and a representative heatmap of activity in the EPM (bottom). **(F)** Distance traveled in the EPM in stress-naïve (*n* = 32), directly stressed (*n* = 25), and indirectly stressed (*n* = 29) males and females. **(G)** Percent of total time spent exploring the open arms of the EPM in stress-naïve (*n* = 32), directly stressed (*n* = 25), and indirectly stressed (*n* = 29) males and females. Error bars, SEM. ns: *P* > 0.05, *P < 0.05, **P < 0.01, ****P < 0.0001.

We previously showed that exposure to the stress paradigm used in the current work resulted in increased immobility in a forced-swim test months after the stress exposure (Lemos et al., 2012). However, others have argued that this type of stress exposure does not produce enduring depressive- or anxiety-like behavior (Mul et al., 2016). They also made a very reasonable argument that lasting changes in swim immobility could be attributable to learning rather than changes in affect. Therefore, this premise raises the intriguing possibility that stress-related physiological changes could spread in a community even when the individuals who are transmitting the phenotype do not exhibit overt behavioral changes; that is, asymptomatic transmission. Therefore, we used a number of behavioral tests that have been classically used to test depressive- and anxiety-like behavior in rodents. First, we investigated whether stress exposure affects social interaction using the three-chamber social approach assay (Figure 5A) where the relative time that mice interact with a novel mouse vs. a novel object was assessed. Since the neurochemical changes in the indirectly stressed animals likely arose through social interactions, we hypothesized that the performance of this group would be particularly affected in the social approach task. However, when tested 1 week after stress exposure, neither directly nor indirectly stressed mice exhibited sociability deficits when males and females were combined for analysis (two-way RM ANOVA with Bonferroni’s *post hoc* tests; interaction of Stress Pretreatment × Zone, *F*_(2,18)_ = 2.005, *P* = 0.1570; Figure 5B). We also analyzed the breakout of male and female groups and have shown the data for full transparency even though these analyses are somewhat underpowered (*n* = 3–5). As with the combined analysis, we did not observe social deficits in any of the groups in either sex (two-way ANOVA with Holm-Sidak’s *post hoc* tests; males: interaction of Stress Pretreatment × Zone, *F*_(2,12)_ = 1.542, *P* = 0.2534; females: interaction of Stress Pretreatment × Zone, *F*_(2,18)_ = 2.534, *P* = 0.1072; Supplementary Figures S3A,B). Notably, we did not observe sexual dimorphism in this behavior as the direct comparison of sociability exhibited by males vs. females failed to reach significance (two-way ANOVA with Bonferroni’s *post hoc* tests; interaction of Stress Pretreatment × Sex, *F*_(2,15)_ = 0.8526, *P* = 0.4460; Supplementary Figure S3C).

Next, we utilized the TST (Figure 5C) to evaluate depression-like behavior in the form of immobility. When male and female groups were combined for analysis, stress exposure did not affect depression-like behavior compared to stress-naïve controls (one-way ANOVA with Dunnett’s *post hoc* tests; *F*_(2,28)_ = 0.1883, *P* = 0.8294; Figure 5D). Similarly, we did not observe differences in immobility between stressed animals and stress-naïve controls when males and females were evaluated separately (one-way ANOVA with Dunnett’s *post hoc* tests; males: *F*_(2,14)_ = 0.2990, *P* = 0.7462; females: *F*_(2,11)_ = 1.921, *P* = 0.1925; Supplementary Figures S4A,B). We did not observe sexual dimorphism in this behavior as the direct comparison of immobility in males vs. females failed to reach significance (two-way ANOVA with Bonferroni’s *post hoc* tests; interaction of Stress Pretreatment × Sex, *F*_(2,25)_ = 1.804, *P* = 0.1854; Supplementary Figure S4C).

Finally, to assess whether mice exhibit anxiety-like behavior following stress exposure we used the EPM (Figure 5E), where a decrease in open-arm time is characteristic of an anxiety-like state. We found that stress exposure did not affect locomotor activity in the EPM when male and female groups were combined (one-way ANOVA with Dunnett’s *post hoc* tests; *F*_(2,83)_ = 0.3935, *P* = 0.6760; Figure 5F), as well as when males and females were evaluated separately (one-way ANOVA with Dunnett’s *post hoc* tests; males: *F*_(2,40)_ = 0.8016, *P* = 0.4557; females: *F*_(2,40)_ = 0.0733, *P* = 0.9295; Supplementary Figures S5A,B), indicating that all groups explored the apparatus. Combining males and females for analysis, our examination of open arm time revealed that indirectly stressed animals spent 24% of total time in the open arms of the EPM, which was significantly less than the directly stressed mice who spent 38% of total time in the open arms (although neither group were different to stress-naïve mice who spent 30% of total time in the open arms), indicating that indirectly stressed animals exhibited significantly more anxiety-like behavior than mice that were subjected to direct stress (one-way ANOVA with Tukey’s *post hoc* tests; *F*_(2,83)_ = 3.969, *P* = 0.0226; Figure 5G). This effect was driven by males in the indirectly stressed group, who spent significantly less time in the open arms of the EPM (20% of total time) compared to stress-naïve and directly stressed males who spent 38% and 41% of total time in the open arms, respectively (one-way ANOVA with Dunnett’s *post hoc* tests; *F*_(2,40)_ = 4.477, *P* = 0.0176; Supplementary Figure S5C). In contrast to males, direct and indirect stress did not affect anxiety-like behavior in females as compared to stress-naïve controls (one-way ANOVA with Dunnett’s *post hoc* tests; *F*_(2,40)_ = 2.466, *P* = 0.0977; Supplementary Figure S5D). Direct comparison of open arm time between sexes revealed that whereas stress-naïve females spent significantly less time in the open arms of the EPM than stress-naïve males, this sex difference in open arm exploration was normalized by stress exposure, resulting in a significant group by sex interaction (two-way ANOVA with Bonferroni’s *post hoc* tests; interaction of Stress Pretreatment × Sex, *F*_(2,80)_ = 3.240, *P* = 0.0443; main effect of stress pretreatment, *F*_(2,80)_ = 4.230, *P* = 0.0179; main effect of sex, *F*_(1,80)_ = 1.733, *P* = 0.1918; Supplementary Figure S5E). Thus, following stress exposure, we did not observe behavioral differences between males and females despite preexisting sex differences in anxiety-like behavior in stress-naïve animals. Overall, these results demonstrate the absence of rFSS-induced enduring, overt behavioral manifestations despite the robust physiological changes that can be broadcast to other members of the population.

## Discussion

The principal findings reported here are threefold: (1) the regulation of dopamine by CRF in the NAc of stress-naïve animals is qualitatively similar between sexes, (2) stress exposure can engender a loss of this regulation in the absence of any overt behavioral manifestations in both sexes, and (3) this stress-induced neurochemical change can be socially transmitted to animals not directly exposed to the primary stressor. These findings support the notion that a stress-related physiological adaptation can be transmitted between members of a population, even when the “infectious” individual does not exhibit overt symptoms of the stress experience.

Concerning potential sexual dimorphism, we have been cautious in asserting the absence of qualitative sex differences in the regulation of dopamine by CRF, without excluding the possibility of small quantitative differences. In NAc brain slices from stress-naïve males and females, we observed that bath application of CRF significantly increased evoked dopamine release relative to vehicle. Although 100 nM CRF resulted in a more robust increase in NAc dopamine release in males than females, this comparison did not reach statistical significance. The vehicle-controlled analysis was sufficiently powered (1 − *β* = 0.80, *α* = 0.05) to observe effect sizes greater than *f* = 0.47. Similar outcomes between sexes were also observed in how stress altered CRF regulation of dopamine and its social transmission. This absence of robust sex differences is in stark contrast to CRF function on some other neural processes where both quantitative and qualitative differences across sexes have been demonstrated (Bangasser et al., 2010; Valentino et al., 2013).

One week after rFSS exposure, we did not observe changes in any of the behavioral metrics tested, specifically, social interaction, depression-like behavior, and anxiety-like behavior. While this testing does not comprehensively rule out every potential behavioral consequence of stressor exposure, it does demonstrate that the classic behavioral phenotype of pathological stress was not present in these individuals. These data corroborate a previous report, which found that male C57BL/6 mice did not exhibit anhedonia (sucrose preference), depression-like behavior (TST), or anxiety-like behavior (OF test) when assessed about a month after rFSS exposure (Mul et al., 2016). The one test in this previous study where enduring behavioral effects were observed was the forced swim test. This finding is similar to work from our laboratory (Lemos et al., 2012). However, it is important to note that shorter latencies to immobility when introduced to the FSS apparatus with repeated exposure can be a learned adaptive phenomenon, confounding the ability to infer depression-like behavior (Mul, 2018; Molendijk and de Kloet, 2019). With that in mind, it appears that the long-term behavioral consequences of stressor exposure were relatively benign. Despite this apparent absence of behavioral manifestation, we replicated our previous finding that the regulation of dopamine release by CRF in the NAc is ablated following rFSS (Lemos et al., 2012). An even more surprising outcome was that this neurochemical adaptation was socially transmitted from the stressor-exposed animals. Thus, while an enduring behavioral phenotype indicative of pathological stress was not present in individuals following the aversive rFSS experience (i.e., they would be considered largely asymptomatic), they robustly transmitted physiological adaptations engendered by the experience.

One possible mode of inter-animal transmission is via secretion of alarm pheromones, as previously demonstrated (Sterley et al., 2018). However, it is unclear whether the animals that were not directly exposed to rFSS experienced stress while cohabiting with the direct-stress conspecifics, or whether they developed the neurochemical change without an aversive experience. We previously found that the loss of CRF regulation of dopamine following direct rFSS exposure is dependent upon glucocorticoid receptor activation during stress exposure (Lemos et al., 2012). However, a similar test on the indirectly stressed animals is more challenging because of the temporal properties of behavioral exposure. Because the cohabitation of the conspecifics in the indirect stress paradigm extends for 7 days, rather than discrete episodes of swim stress on 2 days, glucocorticoid antagonism over that entire time window would interrupt normal behaviors.

In humans, stress transmission is a recognized and ubiquitous phenomenon that pervades all domains of social organization: it can occur on a micro-level (between individuals or within a family unit), meso-level (within a city or statewide community), and macro-level (within a national population or globally; Boroson, 2011). The current work demonstrates that, in a controlled experimental system, stress-related phenotypes can penetrate the population through social transmission in a manner that is undetected through behavioral observations. This process underscores the pervasive nature of stress on our society.

## Data Availability Statement

All datasets presented in this study are included in the article/Supplementary Material.

## Ethics Statement

The animal study was reviewed and approved by University of Washington Institutional Animal Care and Use Committee.

## Author Contributions

The research was conceptualized by JS, JL, CC, and PP. Experiments were designed by JS, BL, CC, and PP and conducted by JS. JS and PP wrote the manuscript with input from all authors.

## Conflict of Interest

The authors declare that the research was conducted in the absence of any commercial or financial relationships that could be construed as a potential conflict of interest.
