# Supplementary material for: Insidious Transmission of a Stress-Related Neuroadaptation
Source: Front Behav Neurosci. 2020 Oct 5;14:564054. doi: 10.3389/fnbeh.2020.564054 (PMC7571264; doi:10.3389/fnbeh.2020.564054)
Supplement: Supplementary file 1 [file Presentation_1.pdf]

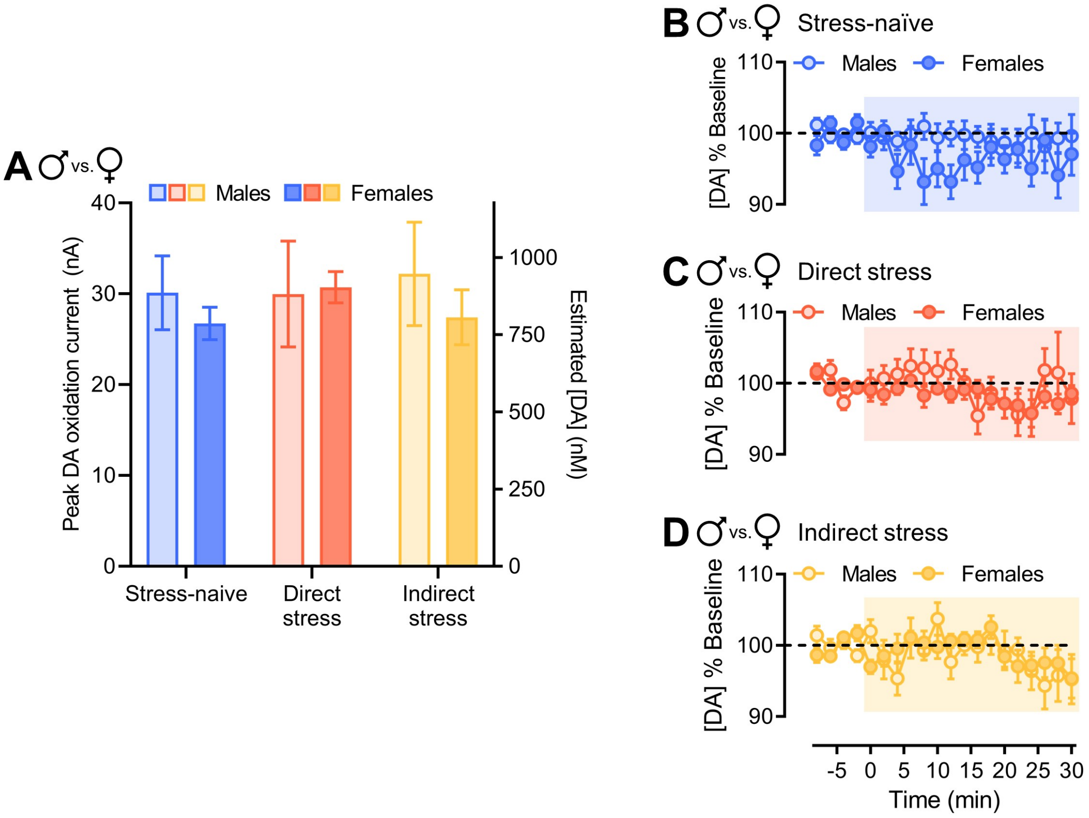

FIGURE S1 | There are no baseline or vehicle-induced differences in evoked NAc dopamine release across treatment groups and sexes. (A) Mean peak amplitude (left y-axis) and estimated concentration (right y-axis) of baseline dopamine release in stress-naïve (n = 20 males, 28 females), directly stressed (n = 10 males, 15 females), and indirectly stressed (n = 10 males, 22 females) animals. (B) Baseline-normalized mean peak amplitude of electrically evoked dopamine release over time in response to vehicle application to NAc slices collected from stress-naïve males (n = 9) and females (n = 10). (C) Baseline-normalized mean peak amplitude of electrically evoked dopamine release over time in response to vehicle application to NAc slices collected from directly stressed males (n = 5) and females (n = 5). (D) Baseline-normalized mean peak amplitude of electrically evoked dopamine release over time in response to vehicle application to NAc slices collected from indirectly stressed males (n = 4) and females (n = 8). Error bars, SEM.

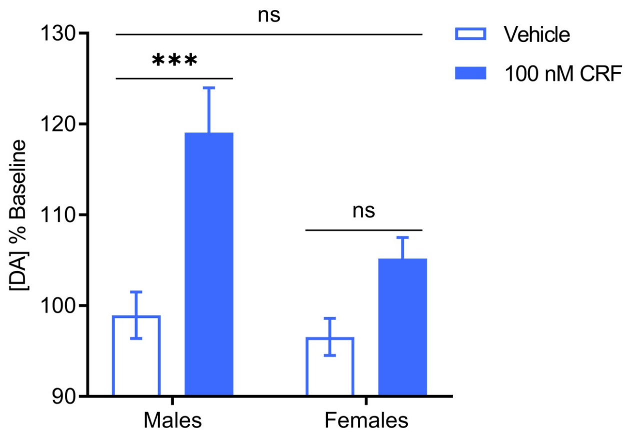

FIGURE S2 | Effects of 100 nM CRF on NAc dopamine transmission is not significantly different between males and females. Baseline-normalized mean peak amplitude of dopamine release 20-30 minutes after vehicle or 100 nM CRF application to NAc slices collected from stress-naïve males (vehicle: n = 9, 100 nM CRF: n = 11) and females (vehicle: n = 8, 100 nM CRF: n = 10). Error bars, SEM. NS P>0.05, ***P<0.001 vs. vehicle.

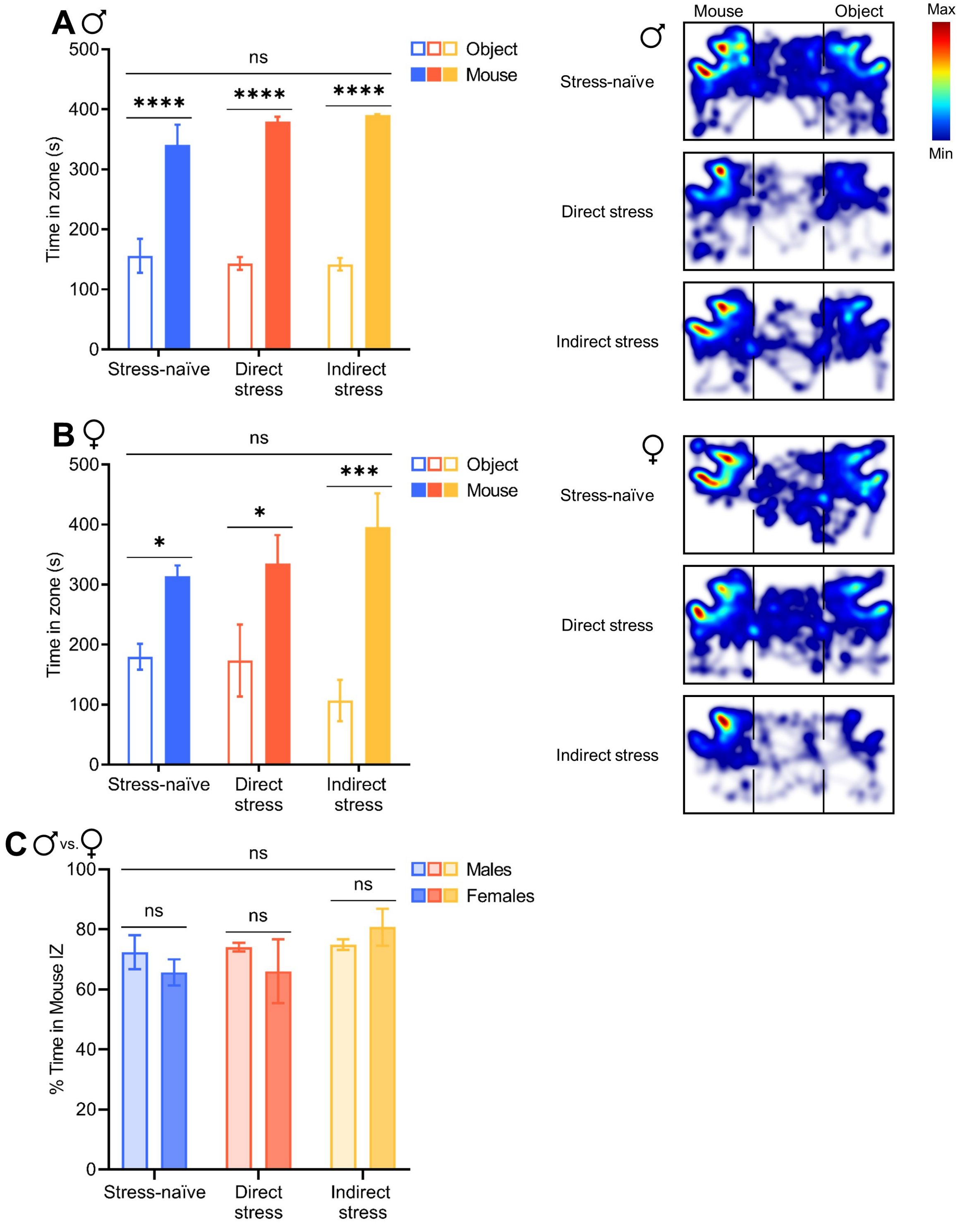

FIGURE S3 | Direct and indirect stress do not affect social interaction one week after stress exposure (A) Amount of time spent in the novel object and novel mouse zones in the three-chamber social approach assay in stress-naïve, indirectly stressed, and directly stressed males ($n = 3$, left) and representative heatmaps of activity in this assay (right). (B) Amount of time spent in the novel object and novel mouse zones in the three-chamber social approach assay in stress-naïve ($n = 5$), indirectly stressed ($n = 3$), and directly stressed ($n = 4$) females (left) and representative heatmaps of activity in this assay (right). (C) Comparison of the percentage of total interaction zone exploration time (defined as time in novel object interaction zone + time in novel mouse interaction zone) that was spent in the novel mouse interaction zone in stress-naïve ($n = 3$ males, 5 females), indirectly stressed ($n = 3$ males and females), and directly stressed ($n = 3$ males, 4 females) males and females. Error bars, SEM. NS $P>0.05$, *$P<0.05$, ***$P<0.001$, ****$P<0.0001$.

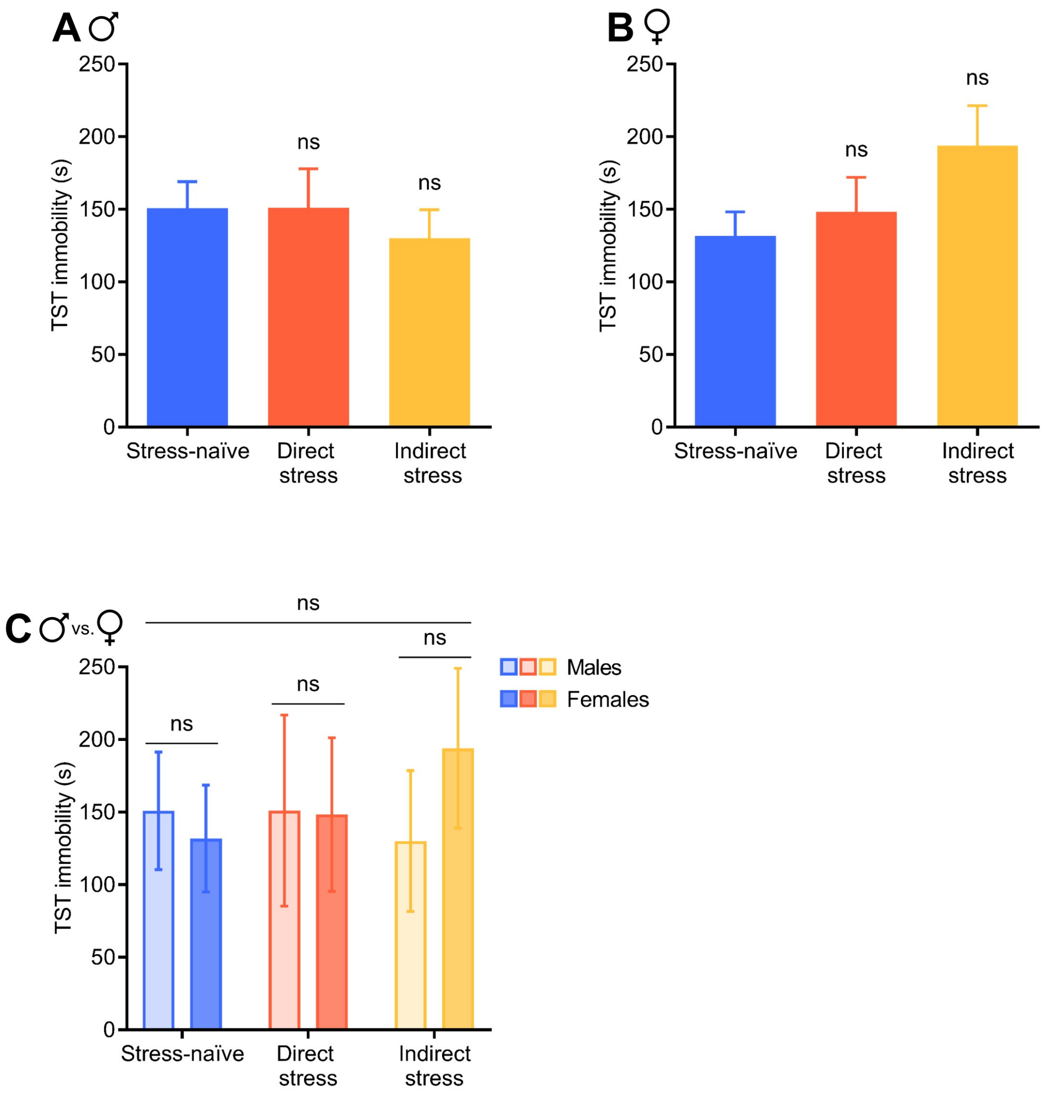

FIGURE S4 | Direct and indirect stress do not affect depression-like behavior one week after stress exposure **(A)** Cumulative time spent immobile for the duration the tail suspension test in stress-naïve ($n = 5$), directly stressed ($n = 6$), and indirectly stressed ($n = 6$) males. **(B)** Cumulative time spent immobile for the duration the tail suspension in stress-naïve ($n = 5$), directly stressed ($n = 5$), and indirectly stressed ($n = 4$) females. **(C)** Cumulative time spent immobile for the duration the tail suspension test in stress-naïve ($n = 5$ males and females), directly stressed ($n = 6$ males, 5 females), and indirectly stressed ($n = 6$ males, 4 females) males and females. Error bars, SEM. NS P>0.05.

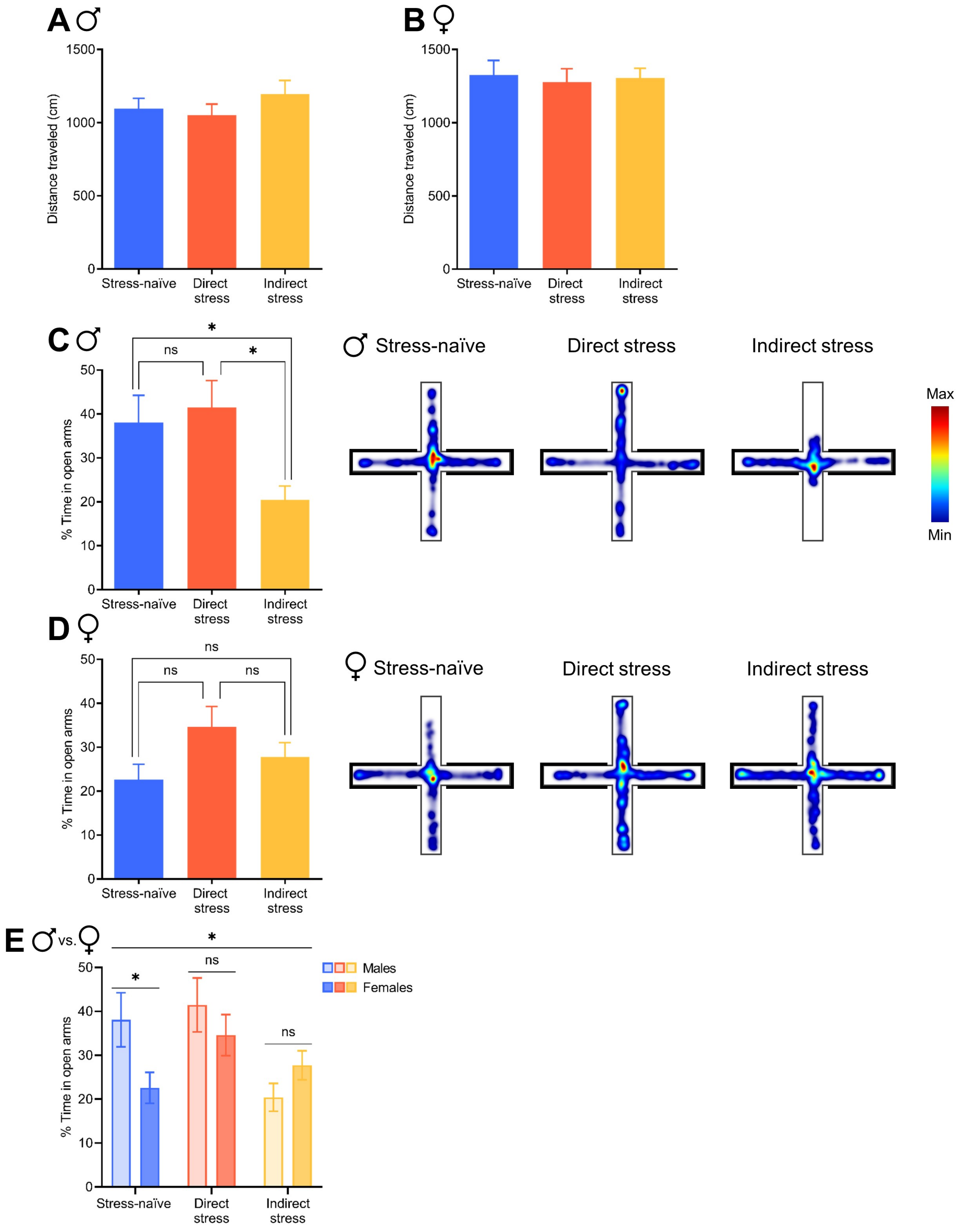

FIGURE S5 | Direct and indirect stress differentially affect anxiety-like behavior in males and females one week after stress exposure **(A)** Distance traveled in the elevated plus maze in stress-naïve (*n* = 16), directly stressed (*n* = 12), and indirectly stressed (n = 15) males. **(B)** Distance traveled in the elevated plus maze in stress-naïve (*n* = 16), directly stressed (*n* = 13), and indirectly stressed (*n* = 14) females. **(C)** Percent of time spent exploring the open arms of the elevated plus maze in stress-naïve (*n* = 16), directly stressed (*n* = 12), and indirectly stressed (*n* = 15) males (left) and representative heatmaps of activity in the EPM (right). **(D)** Percent of time spent exploring the open arms of the elevated plus maze in stress-naïve (*n* = 16), directly stressed (*n* = 13), and indirectly stressed (*n* = 14) females (left) and representative heatmaps of activity in the EPM (right). **(E)** Comparison of percentage of total time spent exploring the open arms of the elevated plus maze in stress-naïve (*n* = 16 males and females), directly stressed (*n* = 12 males, 13 females), and indirectly stressed (*n* = 15 males, 14 females) males and females. Error bars, SEM. NS P>0.05, *P<0.05.
